# Direct effects of physical training on markers of bone metabolism and serum sclerostin concentrations in older adults with low bone mass

**DOI:** 10.1186/s12891-016-1109-5

**Published:** 2016-06-08

**Authors:** Gabriella Császárné Gombos, Viktória Bajsz, Emese Pék, Béla Schmidt, Eszter Sió, Bálint Molics, József Betlehem

**Affiliations:** University of Pécs, Faculty of Health Sciences, 33 Landorhegyi Road, Zalaegerszeg, 8900 Hungary

**Keywords:** Low bone mass, Exercise, Walking, BALP, CTX, Sclerostin

## Abstract

**Background:**

Both gravitational loading and the forces generated by muscle contraction have direct effects on serum markers of bone metabolism. The object of this study was to examine the direct effects of a single session of resistance exercise or walking on biochemical markers of bone metabolism in participants with low bone mass.

**Methods:**

A total of 150 otherwise healthy female subjects (mean age = 59.1 ± 7.1 years) diagnosed with osteoporosis or osteopenia were randomly allocated to either a resistance exercise group (RG; *n* = 50), walking group (WG; *n* = 50), or control group (CG; *n* = 50). Changes in bone-specific alkaline phosphatase (BALP), carboxy-terminal cross-linked telopeptide of type I collagen (CTX), and serum sclerostin concentrations were measured before and immediately after a single exercise intervention.

**Results:**

There was no significant change in BALP values in any of the groups. Sclerostin levels increased in the RG and WG, and there was significant difference between the WG and CG after the exercise intervention (*P* < 0.01). In contrast, the changes in CTX concentrations from baseline were significant in the RG (*P* < 0.01) but not in the WG (*P* = 0.11), and there was a significant difference between resistance exercise and walking (*P* < 0.01).

**Conclusions:**

In participants with low bone mass, resistance exercise influenced the serum concentrations of CTX, a marker of bone resorption, but walking did not.

**Trial registration:**

Current Controlled Trials ISRCTN16329455; retrospectively registered on 05/05/2016.

## Background

Biochemical markers of bone metabolism that reflect the cellular activity related to the formation and resorption of bone are useful in monitoring physical activity, and can help us understand the effects of exercise on bone [1]. The specific mechanisms by which physical exercise exerts its effects on bone metabolism have not yet been fully elucidated. “Bone quality” can be determined by several parameters, including the extent of mineralization, the number and distribution of microfractures, the rate of osteocyte apoptosis, and changes in the collagenous bone matrix [2].

The anabolic effect of physical exercise on osseous tissue is related to mechanical effort, although the osteogenic response may also be influenced by other factors [3]. Physical loads associated with exercise impact bone mass and structure by causing dynamic changes to local mechanical conditions, which stimulate resident osteocytes through fluid shifts in their canalicular network. These osteocytes then produce signaling molecules that regulate bone formation and absorption by osteoblast and osteoclasts [4]. Bone tissue has an intrinsic “mechanostat” that regulates functional adaptation to stresses [5]. Bone deformation of 1500–3000 με (physiological overload) induces modeling of the bone cortex, while that of 100–300 με induces bone multicellular unit (BMU) remodeling. Strain below 100 με is associated with bone loss (disuse atrophy), and fractures may occur with pathological overload [5].

Bone metabolism is influenced by several local factors including the receptor activator of nuclear factor ĸ B (RANK) ligand (RANKL) and osteoprotegerin (OPG) cytokine system, inflammatory cytokines, growth factors, bone morphogenetic proteins, transforming growth factor, prostaglandins, colony stimulating factors, and interleukins [6, 7]. RANKL, a product of osteoblasts, lymphocytes, and bone marrow cells, is the most powerful stimulator of osteoclastogenesis [6]. It exerts its activity by binding to RANK on the cell surface of osteoclast progenitor cells and stimulating their differentiation to mature osteoclasts. OPG blocks the binding of RANKL to RANK and regulates bone resorption by inhibiting osteoclast differentiation [8].

Bone mass can be measured well by densitometry; however, it is more difficult to accurately examine bone structure and strength in live tissue. Some substances produced during bone remodeling are specific biochemical markers of bone metabolism. Products of active osteoblasts can serve as markers of bone formation; serum concentrations of these markers reflect osteoblast function during specific phases of bone formation [9]. Alkaline phosphatase is a ubiquitous tetramer enzyme bound to the cell membrane [9]. In bone, bone-specific alkaline phosphatase (BALP) is present on the surface of osteoblasts and plays an important role in osteoid production and mineralization [10, 11]. Cross-linked telopeptides of type I collagen include C-terminal (CTX, carboxy-terminal cross-linked telopeptide) and N-terminal (NTX, amino-terminal cross-linked telopeptide). These fragments can be produced via different collagen degradation pathways. CTX and NTX are released during the cleavage of cathepsin K [12]. Several assays are available that examine the structural variations in peptide sequences in the CTX [13, 14] and CTX is one of the most sensitive and specific markers of osteoclast-mediated collagen degradation [15]. In addition to osteoblasts and osteoclasts, osteocytes also play an important role in the process of bone remodeling, mainly in its terminal stage, through the production of regulatory factors such as sclerostin (sclerostin gene: SOST). Sclerostin acts on bone surface osteoblasts to inhibit their activity and promote apoptosis [16]. During normal physiological states, the protein sclerostin is differentially expressed in response to mechanical loading, inflammatory molecules such as prostaglandin E2, and hormones such as parathyroid hormone and estrogen [17]. Relatively few studies have researched the effects of physical training on sclerostin levels. Serum concentrations of sclerostin have been shown to be increased by immobilization [18, 19], decreased by physical activity in premenopausal women [20], and to remain steady in physically active postmenopausal women [21].

Weight-bearing and resistance exercises that improve muscle strength are essential for preserving the health of the musculoskeletal system [22]. Coordination, self-assurance, and appropriate muscular strength help to prevent falls and preserve bone mass by stimulating bone formation and reducing bone resorption [23]. Training programs aimed at preserving bone health should incorporate three basic components: 1) impact exercise, such as brisk walking or jogging; 2) strength training with weights; and 3) balance training [24], while lower-impact exercises, such as walking, have minimal effects on density [25]. In contrast to aerobic exercise training, resistance training may have more profound site-specific effects on bone, and progressive resistance training has further advantages in patients with osteoporosis (OP) due to the resulting improvements in muscle strength, mass, and balance [26]. The purpose of this study was to describe the acute response of plasma markers of bone formation (BALP) and resorption (CTX and sclerostin) to a single session of either walking or resistance exercise.

## Methods

### Participants

A total of 150 female subjects diagnosed with osteoporosis/osteopenia (mean age: 58.5 ± 7.5 years) were included in this study. Untreated subjects with recently diagnosed osteoporosis/osteopenia were recruited through a large-scale screening program promoted by the Department of Rheumatology at the Zala County Hospital, Hungary, and implemented through a network of family doctors. The needed sample size was calculated based on expected effect size and study power. There are previous studies in similar contexts with similar interventions which enable exact estimations (*d* = 0.3) [27, 28]. Thus, setting alpha risk to 0.05 and power to 0.08, a sample of 50 per group was needed for two-tailed tests. Nearly 1400 women were examined; 712 were diagnosed as having osteoporosis/osteopenia and 65 of these were excluded because of other health conditions. Thereafter, the women were contacted in random order until 150 had agreed to participate in our study. The participants were randomly assigned to a resistance exercise group (RG; *n* = 50), walking group (WG; *n* = 50), or control group (CG; *n* = 50). Exclusion criteria included (1) any condition influencing calcium and bone metabolism (except dietary calcium and Vitamin D supplementation), (2) ongoing hormone replacement therapy, (3) any known endocrine/metabolic, renal, or hepatic disease (e.g., hypogonadism, hyperthyroidism, hyperparathyroidism, or increased glucocorticoid levels), (4) any physical injury (orthopedic, rheumatologic) hindering the performance of physical activity, (5) osseous fracture of any origin during the previous 6 months, (6) a diagnosis of cardiovascular disease or uncontrolled hypertension, (7) any non-antibiotic medication within the past year, including steroids of any type, thyroid hormones, diuretics, or anticoagulants, and (8) any antibiotic use within the last 6 months.

Ethical approval for this study was issued by the Regional Committee of Science and Research Ethics and the Policy Administration Service of Public Health of Zala County. All participants received written and verbal information regarding the purpose and procedures of the study, and all gave their written informed consent before participation. After the study, appropriate antiporotic medication was provided to the participants as required.

### Study design

#### Resistance exercise group (RG)

The mean age of the participants in the RG was 60.2 ± 6.9 years. Their exercise session included 8 min of dynamic warm-up consisting of exercises requiring movements of large muscle groups and major joints that incorporated small impacts with the ground (steps, combinations of steps, hopping, stretching) [24]. The main part of the session comprised approximately 30 min of exercises that included muscle-strengthening and core stabilization elements. A list of the exercises is provided in Table 1. All exercises were performed as a continuous set; this was followed by a 2-min rest period between sets, and three sets were performed. At the end of the session an 8 min cool down consisting of walking and static and dynamic stretches was performed [24]. The participants familiarized themselves with the exercises prior to participating in the study session.

**Table 1:** Exercises performed by the resistance exercise group during the main part of their exercise session

Starting position	Description of the exercise	Number of repetitions in 1 set
Lying supine with feet on the ground 10 cm apart, arms at sides with palms down.	Shoulder bridge – The pelvis is slowly lifted with tense abdominal and gluteal muscles. This position is held and the knees are alternately extended. The pelvis is then slowly lowered.	4×/side
Lying supine with the hips and knees flexed at 90°	One leg with flexed knee is lowered towards the ground while the contralateral arm is raised vertically. This position is held 2 s and then the exercise is repeated with the opposite limbs.	4×/side
Kneeling position, elbows and forearms on the floor, knees under the hips, trunk stabilized, spine and pelvis stable.	Swimming - The contralateral arm and leg are simultaneously raised until level with the trunk. The exercise is then repeated with the opposite limbs.	4×/side
Static forearm plank.	One leg is raised with the knee extended (lumbar spine in neutral), the position is held for 2 s and then the leg is slowly lowered. This is then repeated with the opposite leg.	4×/side
Static low forearm plank.	The pelvis in held in a raised position for 2 s and then lowered.	6x/side
Sitting on a physioball, legs apart, soles on the ground, hands holding a green band.	The participant leans forward from the hip to 45° with shoulders at approximately 150° flexion while stretching the band. This position is held for 2 s and then the participant slowly returns to the starting position.	8x
Standing straight with feet hip width apart and a ball between the back and a wall.	Ball squatting against a wall – The participant leans into the ball and squats until the thighs are parallel to the ground. The arms are raised and this position is held for 2 s.	8x
Standing with feet hip width apart and tense trunk muscles.	Backward lunge – The knees are bent to a lunge position, which is held for 2 s before returning to the starting stance.	4×/side
Standing with 1 kg weights in both hands.	The participant performs a forward lunge while abducting the arms to 90° and then returns to the starting position.	4×/side
Standing with feet hip width apart and arms straight in front.	The participant steps right/left and lowers into a side lunge before returning to the starting position.	4×/side

#### Walking group (WG)

The mean age of participants in the WG was 58.7 ± 6.3 years. Their exercise session consisted of moderate intensity brisk walking (approximately 3–6 metabolic equivalents) to a rhythm (100 steps/min) provided by a metronome. In this rhythm, they walked continuously for 46 min outdoors on even ground (wearing training shoes). The subjects familiarized themselves with the walking style prior to participating in the study session.

#### Control group (CG)

The mean age of participants in the control group was 57.8 ± 8.4 years. These women did not receive any intervention.

### Laboratory tests

Serum samples were collected and concentrations of markers of bone metabolism were measured at baseline and immediately after the interventions. Several assistants performed sample collection simultaneously to ensure rapid collection within 0–5 min after the exercise sessions. All baseline blood samples were collected between 8:00 and 8:30 AM to minimize circadian effects. Prior to the interventions the participants refrained from exercising for 24 h and fasted overnight. BALP was measured after lectin precipitation using a photometric assay. Test was performed on an Olympus AU 480 Chemistry Analyzer (Brea, USA). CTX was measured by an electrochemiluminescence immunoassay [29, 30]. The reagents for CTX analysis were purchased from Roche (Mannheim, Germany), and measurements were made on a Cobase 411 immunochemical analyzer (Mannheim, Germany). Serum sclerostin (SOST) concentrations were determined by qualitative sandwich enzyme-linked immunosorbent assay (Biomedica, Vienna, Austria). The intra-assay CV was 5–6 %, the inter-assay CV was 2–6 %.

### Physiotherapist-conducted interviews

Participants completed a physiotherapist-administered questionnaire specifically designed to collect demographic data and medical history for this study. Previous and existing diseases (e.g., hypertension, heart disease, bronchial asthma, arthritis, surgical interventions, and autoimmune diseases), medications and supplements (such as Vitamin D), lifestyle factors (sports participation, diet, smoking, and alcohol and coffee consumption), and current physical activity level were determined using the international physical activity questionnaire (IPAQ) [31]. The height of the participants was measured on a Seca medical stadiometer (Birmingham, UK) and their weight was checked on an Omron digital scale (Osaka, Japan).

### Data analysis

All statistical analyses were performed using SPSS (IBM SPSS, Inc., Version 20.0, 2011). Results are expressed as means and standard deviations. Data were checked for normality of distribution by the Kolmogorov-Smirnov test. Levene’s test was used to test homoscedasticity. For the clinical parameters, paired t-tests were used to determine the significance of differences in continuous variables. A two (time) by three (intervention) analysis of variance (ANOVA) with repeated measures was conducted to explore between- and within-group differences and Scheffe post hoc analysis was used to determine the significance of all possible comparisons. The α-level was set at 0.05 for all analyses.

## Results

### Participant characteristics

Participant characteristics are presented in Table 2. There were no significant baseline differences between the groups. The BMI values of the participants ranged from 14.8 to 39.2, with a mean BMI of 27.0 ± 5.4. The participants’ T-scores varied between −4.7 and −1.0 (mean: −2.1 ± 0.8); 38 participants had osteoporosis and 112 had osteopenia. T-scores did not significantly differ between the groups (*P* = 0.31).

**Table 2:** Baseline characteristics of the participants

Variables	RG (*n* = 50)	WG (*n* = 50)	CG (*n* = 50)	*P* value
Age, years	60.2 ± 6.9	58.7 ± 6.3	57.8 ± 8.4	0.15
Height, cm	162.6 ± 9.8	159.6 ± 6.4	161.7 ± 5.0	0.06
Weight, kg	69.7 ± 11.8	72.7 ± 14.8	69.5 ± 13.0	0.41
BMI, kg/m^2^	26.3 ± 5.4	27.2 ± 6.1	28.1 ± 3.9	0.24
T-score	−2.2 ± 0.7	−1.9 ± 0.9	−2.1 ± 0.7	0.31
PTH, pmol/L	5.0 ± 1.6	5.7 ± 2.6	4.9 ± 1.7	0.21
25-OH-D, nmol/L	25.8 ± 11.2	27.1 ± 17.6	26.8 ± 16.0	0.16
Resting heart rate, /min	75 ± 6.2	74 ± 7.1	76 ± 4.7	0.38
Number that occasionally consume alcohol, %^a^	19 (38 %)	16 (32 %)	12 (40 %)	0.43
Number that consume coffee, %^a,^ ^b^	31 (62 %)	37 (74 %)	23 (76 %)	0.21
Smokers, %^a^	5 (10 %)	6 (12 %)	3 (10 %)	0.43
Physical activity, kcal/week^c^	3075 ± 1546	3150 ± 1687	3190 ± 1543	0.54

Values are means ± SD

Abbreviations: *RG* resistance exercise group, *WG* walking group, *CG* control group, *BMI* body mass index, *PTH* parathyroid hormone, *25-OH-D* 25-hydroxy vitamin D

^a^As assessed by baseline questionnaire (prospective participants who consumed more than the occasional alcoholic drink were ineligible for this study)

^b^Minimum 1 cup of strong black coffee daily

^c^From the International Physical Activity Questionnaire

### Bone biomarkers

Baseline and post-intervention serum BALP, CTX, and sclerostin concentrations for the RG, WG, and CG are shown in Table 3. Baseline BALP concentrations ranged from 15.8 to 71.0 % (mean: 41.8 ± 9.6), CTX levels ranged from 30.0 to 685.0 pg/mL (mean: 269.8 ± 127.4), and sclerostin levels ranged from 7.2 to 69.4 pmol/L (mean: 24.4 ± 11.5) for all participants. Post-intervention BALP concentrations did not significantly differ from baseline values, and there were no significant between-group differences in the change in the means from baseline to post-intervention (*P* = 0.21). The RG and CG both experienced only a non-significant rise from baseline in serum sclerostin concentrations, while the WG experienced a significant increase in serum sclerostin. Repeated measures analysis revealed significant group x time interactions (*P* < 0.001). Paired sample t-tests revealed a significant change in CTX concentrations from baseline only in the RG, and repeated measures ANOVA revealed a significant main effect of the type of the physical activity (*P* < 0.01). We compared the osteoporotic and osteopenic participants to determine if there were any differences in the CTX changes from baseline after our interventions; RG participants who had osteoporosis experienced a significant decrease in CTX concentrations post-intervention (*P* < 0.01), but CTX concentrations did not change in participants in the WG or CG (*P* > 0.05).

**Table 3:** BALP, CTX and sclerostin concentrations in the RG, WG, and CG at baseline and post-intervention

Variables	RG (*n* = 50)	WG (*n* = 50)	CG (*n* = 50)
BALP			
Baseline [%]^a^	41.7 ± 12.8	41.8 ± 7.6	42.2 ± 10.4
Post-intervention [%]^a^	41.8 ± 12.0	42.1 ± 8.4	42.1 ± 10.2
Change [%]^b^	+0.1 ± 5.0	+0.9 ± 2.6	−0.14 ± 0.57
* P* value	0.33	0.05	0.19
ANOVA	*P* = 0.21		
Post hoc tests	*P*_RG − CG_ = 0.90; *P*_RG − WG_ = 0.47; *P*_WG − CG_ = 0.23		
CTX			
Baseline [pg/mL]	303.6 ± 156.8	247.3 ± 106.2	259.1 ± 110.2
Post-intervention [pg/mL]	276.4 ± 143.6	253.9 ± 107.5	256.7 ± 111.2
Change [pg/mL]^b^	−27.2 ± 33.5	+6.6 ± 30.7	−2.4 ± 14.6
* P* value (paired t)	<0.01	0.11	0.37
ANOVA	*P* < 0.01		
Post hoc tests	*P*_RG − CG_ < 0.01; *P*_RG − WG_ < 0.01; *P*_WG − CG_ = 0.20		
SCLEROSTIN			
Baseline [pmol/L]	26.8 ± 14.0	23.6 ± 10.0	24.0 ± 8.8
Post-intervention [pmol/L]	29.8 ± 15.7	29.9 ± 10.8	24.2 ± 8.8
Change [pmol/L]^b^	+2.9 ± 8.6	+6.3 ± 12.1	−0.2 ± 8.7
* P* value	0.08	<0.01	0.49
ANOVA	*P* < 0.01		
Post hoc tests	*P*_TG − CG_ = 0.1; *P*_TG − WG_ = 0.11; *P*_WG − CG_ < 0.01		

Abbreviations: *BALP* bone specific alkaline phosphatase, *CTX* C-terminal telopeptide of type-I collagen, *RG* resistance exercise group, *WG* walking group, *CG* control group

^a^BALP expressed as a proportion of the total value of serum ALP

^b^Difference between baseline and post-intervention data

## Discussion

### Direct effect of physical activity on CTX and BALP concentrations

Elevated levels of CTX indicate increased bone resorption and excessive bone turnover [10]. In our study, both resistance exercise and walking induced only a minimal change in BALP, a marker of osteogenesis; however, CTX decreased significantly in the participants who performed resistance exercise, but not in those in the WG. This difference in CTX response was statistically significant, indicating that a single session of resistance exercise reduces bone resorption while a single session of walking does not. This result differs from what would be expected based on previous reports in the literature. According to Banfi et al., exercises performed for a short time are insufficient to change the concentrations of biochemical markers of bone metabolism. In addition, markers of osteogenesis have been reported to be more sensitive than markers of bone resorption to external stresses, and although stimulation of osteoblast and/or osteoclast function is exercise-dependent the osteoblast/clast response to exercise is not immediate [32]. It has been reported that BALP expression by osteoblast precursors transiently increased from baseline 30 min after in vitro mechanical stimulation, before returning to prestimulation levels 24 h later [33, 34]. Significant increases in BALP concentrations have also been shown at the 30th and 50th minutes of cycling, or at the end of the cycling session [27, 28]. All changes in BALP returned to baseline within 20 min after exercise [35]. In the present study, resistance exercise induced an immediate change in CTX and sclerostin levels, but BALP concentrations did not respond to the exercise stimulus. The correlation between bone strength and age-dependent musculoskeletal loading in women has not been fully elucidated, but exercises that increase bone density in young women seem to be ineffective in older adults [36].

Previous studies have reported the short-term effects of different physical activities, including walking [37, 38], outdoor jogging [39], aerobic exercise [28, 40, 41], resistance training [42, 43] and cycling [27, 44, 45], on bone turnover. Gomes-Cabello et al. summarized the effects of different training programs on bone turnover in a systematic review, and found that walking only modestly increased the loads on the skeleton above those caused by gravity; thus, this type of exercise does not effectively prevent osteoporosis in older adults. Resistance exercise/strength training acts as a powerful stimulus to improve and maintain bone mass during the ageing process. Multi-component exercise programs that include strength, aerobic, and high impact and/or weight-bearing exercise, as well as whole-body vibration alone or in combination with exercise, may help to increase, or at least prevent, the decline in bone mass that occurs with ageing, especially in postmenopausal women [46].

Previous studies have reported that serum BALP concentrations increased [27, 28, 47], decreased [42, 48], or remained unchanged [37, 38] after physical activity. The association of changes in CTX concentrations with different types of physical activity has been similarly variable [27, 37, 41, 49]. Thus, it is clear that the response of biochemical markers of bone metabolism to exercise depends on the type and intensity of the exercise performed. Bone mass is the net product of bone formation and resorption, which are tightly regulated by the equilibrium between endogenous/exogenous factors [50]. Biochemical markers of bone formation and resorption are very responsive to physical exercise, but their response varies depending on the type of exercise. Both gravitational loading and the forces generated by muscle contraction play important roles in stimulating the skeletal response to mechanical loading [51].

### Physical activity and sclerostin levels

Normal sclerostin concentrations vary with age and gender. In premenopausal and postmenopausal women normal serum sclerostin concentrations are 24.6 ± 5.7 pmol/L and 30.3 ± 8.8 pmol/L, respectively. In this study, the mean overall sclerostin concentration before exercise was 24.8 ± 11.5 pmol/L. Serum sclerostin levels increase with age in both genders, probably contributing to the age-dependent decrease in bone formation [52].

Situations associated with bone resorption are accompanied by increases in serum sclerostin concentrations. Ardawi et al. demonstrated that even a small increase in physical loading induced a significant decrease in serum sclerostin concentrations and a concomitant increase in markers of bone formation [20]. Spatz et al. confirmed that the mineral content of bone significantly decreased after 90 days of bed rest, and this change was accompanied by a significant increase in serum sclerostin levels [18]. Similarly, postmenopausal women who became immobile as a result of stroke had significantly higher serum sclerostin concentrations than controls; this was accompanied by a decreased bone stiffness index determined by quantitative ultrasound [19]. Lombardi et al. studied athletes and found higher sclerostin levels in females than in males; in addition, sclerostin concentrations were higher in males participating in weight-bearing disciplines than in those who participated in non-weight-bearing sports [50]. Sheng et al. studied healthy postmenopausal women and found significantly higher serum sclerostin levels in those without OP than in those with OP [53]. Xu et al. found a positive correlation between serum sclerostin and lumbar spine bone mineral density and no significant association of serum sclerostin with age or body mass index in postmenopausal osteoporotic women [54]. In the present study, serum sclerostin concentrations increased by 11 and 26 % in participants in the RG and WG, respectively. Bone tissue is mechanosensitive [55]. After a mechanical stimulus, sclerostin secreted by osteocytes travels through the osteocyte canaliculi to the bone surface where it binds to the coreceptors LRP5 and LRP6 (low-density lipoprotein receptor-related protein 5 and 6) thus preventing Wnt (Wingless-type mouse mammary tumor virus integration site family member) signaling and thereby reducing osteoblastogenesis and bone formation [56]. Bone resorption begins immediately after mechanical loading and at the same time, but to a lesser extent, bone formation also begins. Of the parameters that we studied, sclerostin responded the most quickly to mechanical loading. Treatments that inhibit sclerostin may be a promising targeted therapy for bone loss of various origins. Both of the types of physical activity that we studied significantly increased serum sclerostin levels. An antibody that targets sclerostin, decreasing endogenous levels of sclerostin while increasing bone mineral density, is currently in phase-III clinical trials [17]. Thus, if a sclerostin-inhibiting therapy is introduced, the medication will conceivably be more effective when applied after physical exercise.

The strength of the present study was that it examined the direct effects of a single resistance exercise or walking session on sclerostin and biochemical markers of bone metabolism. Its further positive features include the homogeneity of the groups, as well as the careful assessment and exclusion of underlying diseases, medications, hormone replacement therapies, and other confounding circumstances. However, our study also had a number of limitations. It analyzed only the short-term effects of the exercises. Participants had wide age and T-score ranges, both of which have been reported to influence bone turnover [23]. The intensity of the exercise was not well adapted to the participants; a higher intensity may have had more effect on BALP values. In addition, we did not measure serum osteocalcin concentrations. Although both osteocalcin and BALP are osteoblast-specific proteins that are used as markers of bone formation, they are secreted during different phases of osteoblast development [57]. BALP is expressed by newly differentiated osteoblasts, whereas osteocalcin is secreted by mature osteoblasts [57]; thus, it may have been useful to include measurement of osteocalcin concentrations in our analysis.

## Conclusions

Our findings provide insight into the direct effects of resistance exercises and walking on biochemical markers of bone metabolism in older adults with low bone mass. Physical activity of appropriate type, duration, and intensity can influence bone turnover, causing a detectable change in serum concentrations of biochemical markers of bone metabolism and in serum sclerostin levels. The results of the study are consistent with previous reports in the literature indicating that the forces generated by muscle contraction play an important role in stimulating bone resorption, which is evident in the change in CTX concentration after exercise in the RG group. Further studies may uncover the long-term effect of resistance training on sclerostin and Wnt/β-catenin pathway.

## Abbreviations

25-OH-D, 25-hydroxy vitamin D; ANOVA, analysis of variance; BALP, bone-specific alkaline phosphatase; BMI, body mass index; BMU, bone multicellular unit; CG, control group; CTX, carboxy-terminal cross-linked telopeptide of type I collagen; DEXA, dual energy x-ray absorptiometry; IPAQ, international physical activity questionnaire; NTX, amino-terminal cross-linked telopeptide of type I collagen; OPG, osteoprotegerin; PTH, parathyroid hormone; RANK, receptor activator of nuclear factor ĸ B; RG, resistance exercise group; WG, walking group
